# Inflammatory stress responses and future mental health outcomes in people with type 2 diabetes

**DOI:** 10.1016/j.bbih.2022.100472

**Published:** 2022-05-23

**Authors:** Laura Panagi, Lydia Poole, Andrew Steptoe, Ruth A. Hackett

**Affiliations:** Department of Behavioural Science and Health, Institution of Epidemiology and Health Care, University College London, London, UK

**Keywords:** Type 2 diabetes, Interleukin-6, Laboratory stress testing, Depressive symptoms, Mental health, Quality of life, Longitudinal, Follow-up

## Abstract

**Background:**

Inflammatory dysregulation may be linked with mental health disturbances in people with Type 2 Diabetes (T2D), however no previous studies have examined longitudinal associations between inflammatory stress responses and mental health outcomes in T2D.

**Purpose:**

To better understand the biological mechanisms that might predispose people with T2D to poor mental health in the future.

**Methods:**

At baseline, 140 participants with T2D participated in a laboratory stress testing study (mean age = 64 years). Participants underwent two mental stress tasks and blood was sampled before and up to 45 min post-stress to detect plasma interleukin (IL)-6. The Center for Epidemiological Studies-Depression scale and the Short Form-36 Health Survey were completed at baseline and 7.5 years later. We tested associations between IL-6 stress responses and a) depression symptoms and b) mental health-related quality of life (QoL) at baseline and at follow-up using linear regression analyses adjusting for age, sex, and body mass index (BMI). Results: Up to 66 participants provided follow-up data. In cross-sectional analyses, increased IL-6 stress responses immediately post-task were associated with lower mental health-related quality of life (*B* = −21.73, *p* = 0.005, 95% CI [-36.82, −6.63]) adjusting for age, sex, and BMI. In longitudinal analyses, increased IL-6 stress responses at 45 min post-task were associated with increased depressive symptoms (*B* = 10.31 *p* = 0.048, 95% CI [0.10, 20.51]) and decreased mental health-related QoL (*B* = −21.18 *p* = 0.031, 95% CI [-40.34, −2.02]) adjusting for age, sex, and BMI. The association between the 45-min IL-6 response and depressive symptoms at follow-up was diminished after further adjustment for physical health-related QoL and baseline depressive symptoms (*B* = 10.14, *p* = 0.055, 95% CI [-0.21,20.48]).

**Conclusions:**

This study supports the link between inflammatory stress responsivity and future mental health outcomes in people with T2D. Further research involving a larger sample size is required.

## Highlights

• Inflammatory stress reactivity in people with T2D is linked with mental health.
• Larger IL-6 reactivity in people with T2D is linked with lower quality of life.
• Larger IL-6 reactivity in people with T2D is linked with elevated depressive symptoms.

## 1 Introduction

Type 2 diabetes (T2D) is a progressive metabolic disorder that is prevalent among older adults (International Diabetes Federation, 2019). People with T2D have increased risk of depressive disorders (Rotella and Mannucci, 2013) and adverse mental health-related quality of life (QoL) (Arditi et al., 2019) compared to people without T2D (Clouet et al., 2001).

Inflammation and poor mental health are intertwined (Kiecolt-Glaser et al., 2015). A 2020 meta-analysis showed elevated inflammatory markers in depression patients as compared to healthy controls (Osimo et al., 2020). Supporting a causal pathway, higher inflammatory markers predicted future depressive symptoms (Valkanova et al., 2013) and the administration of inflammatory cytokines increased incident depressive symptoms (Rosenblat et al., 2014). Indeed, people with T2D have increased inflammatory levels and show dysregulated interleukin(IL)-6 (re)activity after laboratory stress compared to participants without diabetes (Steptoe et al., 2014). Specifically, individuals living with diabetes had increased circulating inflammatory levels and showed diminished stress responses as compared to matched controls (Steptoe et al., 2014). However, previous studies examining longitudinal associations between inflammatory stress responsivity and mental health outcomes in the general population or in people with T2D are sparse.

Laboratory stress responses are hypothesised to index how people react to everyday-life stressors. This research strategy is focused on dynamic changes in stress biomarkers from pre-to post-stress, which are not detectable if single/resting measures are taken (Gerin, 2010). Longitudinal associations between inflammatory stress reactivity and health outcomes predominantly show that greater inflammatory reactivity, in terms of its magnitude and/or duration, has negative implications for physical health in the long-term, after controlling for resting inflammatory levels (Brydon and Steptoe, 2005; Steptoe et al., 2016). In another study (Ellins et al., 2008), inflammatory stress responses but not resting levels have been associated with future physical health outcomes, adding value to examining inflammatory stress responsivity (Ellins et al., 2017). IL-6, specifically, is responsive to laboratory stress by showing consistent increases (Panagi et al., 2019; Steptoe et al., 2014) whereas other inflammatory factors have not been found to increase post-stress in people with T2D (Panagi et al., 2019).

Previous laboratory stress testing studies are mostly focused on physical health outcomes in the general population. The purpose of this study is to understand the biological mechanisms that might predispose people with T2D to increased risk of future mental health problems. The effect of IL-6 stress responses on depressive symptoms and mental health-related QoL in people with T2D is assessed.

## 2 Methods

### 2.1 Participants

In 2011/12, 140 individuals aged 50-75 years old with doctor-verified T2D participated in a laboratory stress testing study, the Diabetes Study (Steptoe et al., 2014). The Diabetes Study was powered to detect small to medium effect sizes (δ = 0.32, *p* < 0.05), and at least 125 people needed to be recruited. Participants were recruited from diabetes outpatient and primary care clinics in London. However, the study response rate was difficult to assess as primary care practices differed substantially in their ability to record the number of screened medical records and subsequently, for many practices, the study researchers could not estimate the number of eligible participants or the number of invitation letters sent out before an interested person contacted the study team at University College London (UCL). People who reported a history of coronary heart disease (CHD), inflammatory diseases, allergies, or mood disorders were excluded. These criteria were selected in order to reduce potential interference with biological processes and stress responsivity. It was not possible to exclude obese individuals from this study as obesity is one of the main risk factors for T2D (Guh et al., 2009), and the majority of eligible participants were obese.

In 2019 (7.5 years follow-up on average), all living participants of the Diabetes Study were invited to take part in a postal questionnaire-based study (the Diabetes Follow-up Study). Fully informed written consent was obtained at baseline and follow-up. Ethical approval was granted by the UK National Research Ethics Service (ref 97/0356 for the baseline study and ref 18/ES/0121 for the follow-up study).

### 2.2 Procedure

Participants of the Diabetes Study were requested to avoid anti-inflammatory or antihistamine medication seven days before laboratory testing. Participants were instructed to not undertake vigorous exercise and not consume alcohol from the evening before testing, and to avoid caffeine or smoking for 2 h before the session. Participants who reported colds or other infections were rescheduled.

Participants were tested individually in our light- and temperature-controlled laboratory, in the morning or afternoon. Anthropometric characteristics were first assessed. A venous cannula was then inserted into participants’ forearm and the participant rested for 30 min. During the last 5 min of the resting phase, a blood sample was drawn for the measurement of pre-stress IL-6. Two stress tasks were then administered, and blood was sampled again immediately post-task and 45 min post-task.

Blood samples were centrifuged immediately at 2500 rpm for 10 min at room temperature using ethylenediaminetetraacetic acid tubes. Plasma was removed from the tubes, aliquoted into 0.5 ml portions, and stored at −80° Celsius until later batch analysis. Analysis was carried out using the HS600B Quantikine high sensitivity two-site enzyme-linked immunosorbent assay from R & D Systems (Oxford, UK). The minimum limit of detection for IL-6 was between 0.016 pg/ml and 0.110 pg/ml. The mean intra- and inter-assay coefficients of variation for IL-6 was 7.3% and 7.7%, respectively (R&D Systems, 2022).

### 2.3 Mental stress tasks

Two 5-min tasks were administered in random order, with the participant seated and the experimenter present in the room. The computerised-version of the Stroop colour-word interference task requires successive reporting of target colour words (e.g., green) printed in an incongruous colour. The mirror tracing task requires participants to move a metal stylus to trace a star while looking at the mirror image. When the stylus comes off the star's outer line, a loud noise is emitted indicating that a mistake is counted. Participants were informed that an average person achieves five tracings with minimum mistakes in 5 min. The two tasks are used widely in laboratory stress testing studies as they present important advantages. For example, they have been shown to induce robust biological responses and they seem to stimulate similar appraisals of involvement and engagement from participants across the social gradient (Hamer et al., 2009; Steptoe et al., 2002). Additionally, we previously demonstrated that the two tasks induce significant increases in IL-6 post-stress in T2D participants (Panagi et al., 2019).

### 2.4 Predictor variables: IL-6 stress responses

Two IL-6 mean change/delta (Δ) scores were used as predictor variables, reflecting the mean difference/change in IL-6 from baseline to post-task: immediately post-task minus baseline (ΔIL-6 immediately post-task) and 45 min post-task minus baseline (ΔIL-6 45 min). Higher positive delta scores indicated greater IL-6 increases from baseline to post-task.

### 2.5 Outcome variables

#### 2.5.1 Depressive symptoms

Depressive symptoms were measured using the Centre for Epidemiological Studies-Depression (CES-D) scale (Radloff, 1977) at baseline (Cronbach's α = 0.86) and follow-up (Cronbach's α = 0.61). Items measure affective and somatic dimensions of depression. Items were scored in a Likert-type scale ranging from 0 to 3, resulting in a total score ranging between 0 (no symptoms) to 60 (all 20 symptoms). For longitudinal analyses, a change score (follow-up minus baseline) was created and used as an outcome measure to reflect change in depressive symptoms over time; a larger positive score indicates an increase in depressive symptoms at follow-up.

#### 2.5.2 Mental health-related QoL

The mental health component of the Short Form(SF)-36 Health Survey (Ware and Sherbourne, 1992) was administered to assess mental health-related QoL at baseline (Cronbach's α = 0.78) and follow-up (Cronbach's α = 0.76). This dimension includes subscales relating to limitations due to emotional problems, mental health, social functioning, or fatigue/loss of energy or vitality, all of which were used to calculate an average score. Scores range from 0 to 100, with greater scores indicating better mental health-related QoL. For the longitudinal analyses, a change score (follow-up minus baseline) was created and used as an outcome measure to reflect change in mental health-related QoL over time; a higher positive score indicates better QoL at follow-up.

#### 2.5.3 Other measures considered in analyses

To describe our sample, we provide information on mean glycated hemoglobin levels (HbA1c [measured in blood before stress testing]), medication intake (self-reported and also inspected on the day of testing [yes/no]) including anti-diabetic drugs, insulin or other injectable anti-diabetic medication, anti-hypertensive medication, beta-blockers, cholesterol-lowering drugs, and aspirin, and diabetes duration (self-reported at follow-up). We ran unadjusted linear regression analyses to test for associations between HbA1c and medications with the outcomes of interest, cross-sectionally and longitudinally. Anti-hypertensive medication was cross-sectionally associated with quality of life, but when adding this variable as an extra covariate in the cross-sectional quality of life regression model the pattern of significant results did not change. All other factors showed no significant associations with our outcomes (*p* values ≥ 0.056), hence were not included as covariates in the regression models.

Age (in years), sex (men/women), and body mass index (BMI; objectively measured [kilograms per square metre]) were included as covariates in main regression models. The physical health component of the SF-36 (Ware and Sherbourne, 1992) was included as a covariate in secondary models. It was administered at baseline (Cronbach's α = 0.91) to measure physical functioning, bodily pain, role limitations due to physical health problems, and general health perceptions, all of which were used to calculate an average score. Scores range from 0 to 100, with greater scores indicating better physical health-related QoL.

### 2.6 Statistical analysis

Independent samples *t*-tests for continuous variables and Chi-square tests for categorical variables were conducted to test for differences in sample characteristics between people who were retained in the study and those who were lost to follow-up.

The distribution of IL-6 concentrations was positively skewed (skewness >1 for all IL-6 measurements; kurtosis values 1.57–2.98). Log-n transformation was applied in all analyses (except the descriptive statistics for ease of interpretation). Descriptive statistics were first carried out to describe our sample in terms of glycemic control, medications, and diabetes duration. Descriptive characteristics for baseline and follow-up measures are presented as means (standard deviations) and numbers (percentages). One-way repeated measures analysis of variance (ANOVA) was conducted to test for differences in depressive symptoms and mental health-related QoL between baseline and follow-up.

Associations between IL-6 stress responses and mental health outcomes were examined cross-sectionally and longitudinally using multivariable linear regressions. Separate regression analyses were carried out for each IL-6 change score and for each outcome measure adjusting for age, sex, and BMI. Covariates were chosen based on research indicating their effect on inflammatory stress responses and/or mental health. All covariates were assessed at baseline.

In secondary analyses, significant findings from main regression analyses were further adjusted for the physical health component of QoL and in longitudinal analyses for the baseline mental health measure. In sensitivity analyses, we tested whether the two tasks elicited significant increases in IL-6 for the baseline sample and for the sample retained at follow-up using one-way repeated measures ANOVA. We also tested whether there were significant differences in IL-6 responsivity between the sample who tested in the morning as compared to those tested in the afternoon using mixed model ANOVA.

We carried out complete case analysis based on available data. Variability in missing data across study variables resulted in different numbers for each variable/model. Significance was set at *p* < 0.05. Statistical analyses were carried out using SPSS v27.

## 3 Results

### 3.1 Sample characteristics

**Fig. 1:**
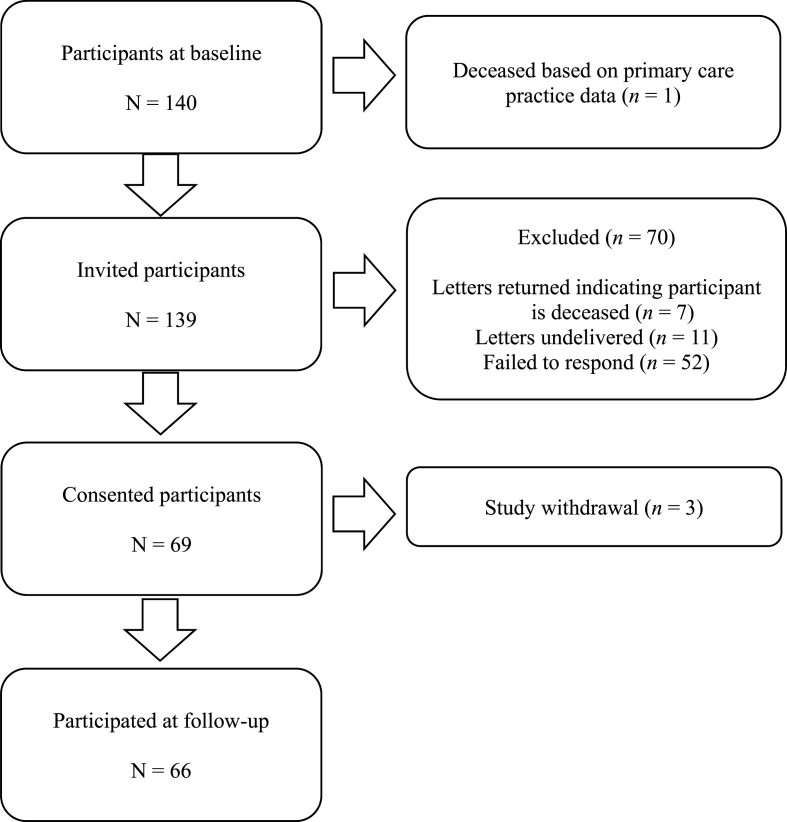
Flow diagram of participants lost to follow-up. N = number; n = number.

Mean HbA1c levels were 7.25% (SD = 1.43) and the mean diabetes duration (as measured at follow-up) was 15.74 years (SD = 7.49). A total of 80.1% of the sample was on oral anti-diabetic drugs and 11% of the sample was taking insulin or other injectable anti-diabetic medication. Also, 70.6% of the sample were taking anti-hypertensive medication, 11.8% of the sample were taking beta-blockers, 77.9% of the sample were taking cholesterol-lowering drugs, and 35% of the sample were taking aspirin. Out of 140 participants of the initial study, 53% (*n* = 74) were lost to follow-up (Fig. 1). Participants with higher IL-6 absolute levels at two time points (pre-task: *t*(119.95) = −2.07, *p* = 0.041, *d* = 0.36, 95% CI [−0.90, −0.02]; 45 min post-task: *t*(97.80) = −2.84, *p* = 0.006, *d* = 0.52, 95% CI [−1.20, −0.21]) were more likely to be lost to follow-up. Participants with elevated depressive symptoms (*t*(122.77) = −2.54, *p* = 0.012, *d* = 0.43, 95% CI [−6.63, −0.82]) and lower mental health-related QoL (*t*(126.30) = 2.06, *p* = 0.041, *d* = 0.35, 95% CI [0.27, 13.07]) were less likely to be retained. No other significant differences were observed between those who were retained and those lost to follow-up (*p* values ≥ 0.487).

Up to 66 participants provided follow-up data (Table 1). When comparing mental health symptoms from baseline to follow-up in the retained sample we observed that depressive symptoms significantly increased (*F*(1.00, 58.00) = 5.43, *p* = 0.023, *η*^*2*^_*p*_ = 0.09) over time (from 9.43 [SE = 0.85] to 11.42 [SE = 1.11]). Mental health-related QoL did not significantly change (*F*(1.00, 65.00) = 0.02, *p* = 0.886, *η*^*2*^_*p*_ = 0.00) over time (from 75.29 [SE = 1.86] to 74.99 [SE = 2.40]).

**Table 1:** *Sample characteristics at baseline (2011/2012; N ≥ 115) and follow-up (2019; N ≥ 60)*.

**Baseline characteristics (2011/2012; N ≥ 115)**	*n* (%) or M ± SD
**Age [years]**	63.71 ± 7.00 *n* = 140
**Sex [women]**	52 (37.1) *n* = 140
**BMI [kg/m**^**2**^**]**	30.75 ± 5.73 *n* = 138
**Pre-task IL-6 [pg/ml]**	2.15 ± 1.30 *n* = 130
**Immediately post-task IL-6 [pg/ml]**	2.18 ± 1.26 *n* = 126
**45 min IL-6 [pg/ml]**	2.30 ± 1.41 *n* = 115
**Depressive symptoms at baseline [score]**	11.86 ± 8.93 *n* = 137
**Mental health-related QoL at baseline [score]**	71.78 ± 19.63 *n* = 139
**Follow-up characteristics (2019; N ≥ 60)**	*n* (%) or M ± SD
**Depressive symptoms at follow-up [score]**	11.42 ± 8.53 *n* = 60
**Mental health-related QoL at follow-up [score]**	74.99 ± 19.52 *n* = 66

*Note*. Complete case analysis was carried out based on available data. IL-6 values are unlogged. BMI = body mass index; IL-6 = interleukin-6; kg/m^2^ = kilograms per square metre; M = mean; min = minutes; N = number; *n* = number, pg/ml = picogram per millilitre; QoL = quality of life; SD = standard deviation.

### 3.2 Cross-sectional associations between IL-6 stress responses and mental health

No significant relationships were found between IL-6 stress responses and baseline depressive symptoms adjusting for age, sex, and BMI (*p* values ≥ 0.184). IL-6 stress responses immediately post-task were significantly associated with baseline mental health-related QoL adjusting for covariates (*B* = −21.73, *p* = 0.005, 95% CI [−36.82, −6.63]), suggesting that those with greater IL-6 stress responses post-task had lower mental health-related QoL. The 45-min response was not associated with mental health-related QoL at baseline (*p =* 0.641).

### 3.3 Longitudinal associations between IL-6 stress and mental health

Elevated IL-6 responses at 45 min post-stress were significantly associated with increased depressive symptoms at follow-up adjusting for age, sex, and BMI (*B* = 10.31 *p* = 0.048, 95% CI [0.10, 20.51]; Table 2). Increased IL-6 stress responses at 45 min post-task were also linked to lower mental health-related QoL at follow-up adjusting for covariates (*B* = −21.18 *p* = 0.031, 95% CI [−40.34, −2.02]; Table 2). IL-6 responses immediately post-task were not significantly associated with depression or QoL at follow-up (*p* values ≥ 0.651).

**Table 2:** *Linear regressions on IL-6 responses (2011/2012) and mental health outcomes (2019)*.

**Model**	Unstandardised Coefficients		95% CI	*p* value
	*B*	SE		
**Dependent variable: Depressive symptoms (N = 47)**				
**45 min IL-6 response [ln]**	10.31	5.06	0.10 to 20.51	**0.048**
**Age [years]**	−0.01	0.17	−0.35 to 0.33	0.969
**Sex [ref. cat.: men]**	1.71	2.19	−2.71 to 6.14	0.439
**BMI [kg/m**^**2**^**]**	0.12	0.19	−0.27 to 0.51	0.544
**Dependent variable: Mental health-related QoL (N = 53)**				
**45 min IL-6 response [ln]**	−21.18	9.53	−40.34 to −2.02	**0.031**
**Age [years]**	−0.42	0.37	−1.16 to 0.33	0.265
**Sex [ref. cat.: men]**	−9.80	4.89	−19.62 to 0.03	0.051
**BMI [kg/m**^**2**^**]**	−0.60	0.43	−1.45 to 0.26	0.167

*Note.* Complete case analysis was carried out based on available data BMI = body mass index; CI = confidence interval; IL-6 = interleukin-6; kg/m^2^ = kilograms per square metre; ln = log-n; min = minutes; *n* = number; ref. cat. = reference category; QoL = quality of life; SE = standard error.

### 3.4 Secondary analyses

The cross-sectional association between the immediate post-task IL-6 response and mental health-related QoL was diminished after adjustment for physical health-related QoL (*B* = −8.00, *p* = 0.159, 95% CI [−19.18, 3.19]). Also, the association between the 45-min IL-6 response and depressive symptoms at follow-up was reduced after further adjustment for physical health-related QoL and baseline depressive symptoms (*B* = 10.14, *p* = 0.055, 95% CI [−0.21,20.48]). The longitudinal association between the 45-min IL-6 response and mental health-related QoL was upheld after further adjustment for physical health-related QoL and baseline mental health-related QoL (*B* = −21.97, *p* = 0.026, 95% CI [−41.19, −2.74]).

### 3.5 Sensitivity analyses

We found a significant effect of time on IL-6 values for the baseline sample (*p* = 0.041), suggesting that the two tasks elicited significant increases in IL-6 from pre-to post-task. In the sample retained in the study, average IL-6 levels increased from pre-task (M = 1.82 [SD = 0.12]) to 45-min post-task (M = 1.93 [SE = 0.13]), albeit effects were not significant (*p* = 0.200). There were no significant differences in IL-6 responsivity between the sample who tested in the morning as compared to those tested in the afternoon (*p* = 0.422).

## 4 Discussion

This study investigated, for the first time, the longitudinal relationship between inflammatory stress responses and mental health outcomes in T2D. Our findings suggest that greater IL-6 stress responses are associated with increased depressive symptoms at follow-up and lower mental health-related QoL at baseline and at follow-up in people with T2D.

This study corroborates findings from a previous cross-sectional study in older participants showing that greater loneliness in women was associated with larger IL-6 stress responses (Hackett et al., 2012). Another laboratory stress study (Aschbacher et al., 2012) with 35 women found that a greater decline in positive affect and cognition during acute stress predicted increases in depressive symptoms one year later and this association was mediated via heightened inflammatory (IL-1b) stress responses 30 min post-stress. Our study adds to previous evidence by demonstrating a temporal relationship between heightened IL-6 stress responses and increased mental health-related symptoms in people with T2D. Notably, a longitudinal association between IL-6 and mental health was only observed with change in IL-6 at 45 min post-task and not with immediate post-task change. Similarly, two previous laboratory stress studies examining physical health outcomes at follow-up demonstrated a stronger effect of the fibrinogen 45-min response versus the immediate response (Ellins et al., 2008, 2017). It is well-established that IL-6 shows delayed increases after stress (Marsland et al., 2017) and we previously found that stress-induced IL-6 levels increase significantly only at 45 min post-stress in this sample (Panagi et al., 2019). Of note, the observed associations may not be specific to T2D but might extend to other inflammatory-related diseases such as CHD obesity, and autoimmune diseases. This hypothesis should be explored in future studies.

The cross-sectional association between IL-6 and mental health-related QoL was not upheld in secondary analyses after further adjustment for the physical health component of QoL. The latter was significant in the secondary model offering the possibility that physical health-related QoL may be a potential mechanism that should be further explored in future studies. The longitudinal association between IL-6 and depressive symptoms was also diminished in secondary analyses, after further adjustment for physical health-related QoL and baseline depressive symptoms. Given the small sample size, it may be that the model was over-adjusted. Indeed, the analysis of depressive symptoms was not upheld in secondary analysis, while the analysis of QoL remained significant. A statistical power issue could account for this finding, since the number of participants with completed CES-D score was 10% less than the number of participants who completed the SF-36. Future studies with larger samples are needed to replicate our findings.

The mechanisms through which IL-6 stress responses and mental health are related are not clear. Hypothetically, inflammatory hyperactivity may be associated with sickness behaviour (Dantzer et al., 2008) that may be reflected in impaired mental functioning in the long-term. Ample evidence supports a direct effect of inflammatory biomarkers on mood and well-being. For example, pre-clinical studies have shown that inflammatory cytokines, including IL-6, act centrally to induce negative mood symptoms (Rosenblat et al., 2014). In animal and human studies, the administration of inflammatory cytokines, such as interferon-γ, increases depressive symptoms (Rosenblat et al., 2014) and fatigue (Majer et al., 2008; Malik et al., 2001). Additionally, two literature reviews indicate that the administration of inflammatory cytokines inhibitors, such as tumor necrosis factor-α antagonists and IL-1 inhibitors, reduce depressive symptoms, anxiety, and fatigue, and increase vitality and mental health-related QoL across different patient populations (Raison et al., 2013; Yadlapati and Efthimiou, 2016). The immune system may also be related to subsequent well-being indirectly via hypothalamic-pituitary-adrenal (HPA) axis activity. Supporting this notion, there is evidence that mood symptoms are subserved by disturbances in interacting inflammatory and neuroendocrine networks (Soczynska et al., 2009). Inflammatory cytokines may initiate, promote, and maintain immune-stimulus-associated HPA reactivity and variations in HPA axis responses have been associated with psychological distress and major depressive disorder (Hurwitz and Morgenstern, 2001). Future studies need to elucidate the precise mechanisms linking IL-6 stress processes with poorer mental health in people with T2D.

This study benefitted from a standard stress testing protocol and its longitudinal design. However, even though the original Diabetes Study was powered, the follow-up study was not (because it had not been planned at the time), and therefore the sample size was decided pragmatically. 54% of the sample was lost to follow-up, increasing the possibility of a type 1 error. From those who were retained, up to 18% had missing post-task IL-6 data due to difficulties with repeated blood sampling. Low response rate was expected given the older age and disease status of our sample. At least eight people were deceased over the follow-up period and 11 invitation letters were undelivered, suggesting that people may have moved houses. Indeed, the London population is highly mobile and some primary care practices closed over the 7.5 years, meaning it was hard to track patients down. And even though participants gave fully informed written consent to contact their primary health care providers and access their medical records, only nine out of 31 practices agreed to take part to the study (after several efforts to contact them), covering 13 out of 69 consented participants. Given the small number of responding practices and due to time constraints, clinical data were not collected for this study. The focus of this study was not on examining a clinical diagnosis of depression but instead the symptoms indicative of psychological distress and potential mood disturbance, hence we did not impose a cut-off on CES-D scores. Increases in depressive symptoms, as seen at follow-up, might be transient, but this is something we did not test in this study. Results of this study were adjusted for potential confounders, but the small sample size restricted the number of covariates included. Participants were recruited from London and most were of white ethnicity therefore these findings may not be generalised to other cohorts. Also, enrollment was limited to people with T2D and no history or previous diagnosis of CHD, inflammatory diseases, allergies, or mood disorders. Even though strict inclusion criteria may minimise the risk of extraneous factors being present, these may also restrict the external validity of the results (Patino and Ferreira, 2018). For example, by restricting the sample to people with T2D and without CHD raises issues about the representativeness of the study, since a large number of older people with T2D (up to 29.1%) have comorbid CHD (Einarson et al., 2018).

In conclusion, this study supports a link between inflammatory stress responses and future mental health in people with T2D. Larger-scale studies are needed to replicate findings.

## Declaration of competing interest

None.
